# Share buybacks: a theoretical exploration of genetic algorithms and mathematical optionality

**DOI:** 10.3389/frai.2023.1276804

**Published:** 2023-11-03

**Authors:** Joerg Osterrieder

**Affiliations:** ^1^Department of High-Tech Business and Entrepreneurship, University of Twente, Enschede, Netherlands; ^2^Bern Business School, Institute of Applied Data Science and Finance, Bern, Switzerland

**Keywords:** share buybacks, genetic algorithms, mathematical optimization, optionality, trading schedules, financial innovation, computational intelligence

## Abstract

This article exclusively formulates and presents three innovative hypotheses related to the execution of share buybacks, employing Genetic Algorithms (GAs) and mathematical optimization techniques. Drawing on the foundational contributions of scholars such as Osterrieder, Seigne, Masters, and Guéant, we articulate hypotheses that aim to bring a fresh perspective to share buyback strategies. The first hypothesis examines the potential of GAs to mimic trading schedules, the second posits the optimization of buyback execution as a mathematical problem, and the third underlines the role of optionality in improving performance. These hypotheses do not only offer theoretical insights but also set the stage for empirical examination and practical application, contributing to broader financial innovation. The article does not contain new data or extensive reviews but focuses purely on presenting these original, untested hypotheses, sparking intrigue for future research and exploration.

**JEL Classification:**

G00.

## 1. Introduction and background

Share buybacks, the practice of corporations repurchasing their own shares from the market, has become a prominent strategy in modern financial management. It serves various purposes, ranging from returning capital to shareholders to managing stock price and optimizing capital structure. This versatile financial tool has attracted extensive scholarly attention, giving rise to a rich body of literature. Pioneering researchers such as Guéant et al. (2015, 2020), Guéant (2017), Masters (2023), Seigne and Osterrieder (2023a), and Osterrieder and Seigne (2023a,d,h), have explored various facets of share buybacks, laying the groundwork for this study.

For a more in-depth understanding of the execution phase of share buy-backs, the collection of papers authored by Seigne and Osterrieder (2023b) serves as the inaugural comprehensive analysis, shedding light on multiple dimensions (Osterrieder, 2023; Osterrieder and Seigne, 2023b,c,e,f,g).

While conventional methods for executing share buybacks are well-researched, the ever-evolving financial landscape and the intricacies of market dynamics necessitate continuous innovation. The challenges that surround share buyback execution have prompted a search for alternative approaches that can transcend traditional boundaries.

Parallel to this need, the emergence of Genetic Algorithms (GAs) represents a significant stride in computational intelligence. These algorithms, inspired by natural selection and evolutionary principles, have shown remarkable versatility in solving complex optimization problems across various domains. The adaptability and efficiency of GAs have made them a tool of choice in sectors that require precise and robust optimization strategies.

The fusion of share buybacks and Genetic Algorithms forms the central premise of this article. This intersection is not merely a convergence of two disparate fields but a deliberate integration aimed at revolutionizing share buyback strategies. By applying Genetic Algorithms to the execution of share buybacks, this study seeks to shed light on uncharted territories, paving the way for unprecedented innovations.

In addition to GAs, the concept of mathematical optionality is explored as a novel approach to optimizing share buyback execution. By viewing the execution as a mathematical problem, new dimensions of analysis and strategic planning are unveiled. This perspective introduces a level of flexibility and adaptability that can potentially enhance the effectiveness of buyback strategies.

Together, these concepts form the core of three innovative hypotheses, each providing a fresh take on share buyback execution. They are designed to spark interest, provoke thought, and inspire further research in the intersection of finance and computational methods.

The article's subsequent sections will delve into the formulation and development of these hypotheses, providing a theoretical exploration grounded in the foundational work of leading researchers. Through this endeavor, the article aims to contribute to the broader discourse on financial innovation, setting the stage for empirical investigations, and fostering collaboration between finance and technology.

In conclusion, this article marks a pivotal step toward a new frontier in financial research, where the synergy of computational intelligence, mathematical optimization, and traditional financial economics can usher in a new era of innovation and efficiency. By presenting original, untested hypotheses, it invites readers to engage with new possibilities and explore paths less traveled in the ever evolving landscape of finance.

## 2. Hypotheses development

In this section, we articulate three hypotheses that build upon the theoretical constructs previously discussed. These hypotheses are designed to extend our understanding of share buybacks by applying novel insights from the domain of Genetic Algorithms and mathematical optimization.

**H1: genetic algorithms and trading schedules**

Our first hypothesis contends that Genetic Algorithms (GAs), with their robust search and optimization capabilities, can successfully replicate empirically observed trading schedules for share buybacks. This hypothesis stems from the adaptability and efficiency of GAs in solving complex problems, proposing that these algorithms can model and simulate the dynamics of trading schedules with high fidelity.

Genetic Algorithms are inspired by the biological process of evolution, encompassing mechanisms such as selection, crossover, and mutation to explore a given search space. In the context of financial markets, trading schedules represent a series of time-based decisions that determine the buying or selling of shares. Replicating such schedules using traditional mathematical approaches can be challenging due to the dynamic, non-linear nature of financial markets. However, GAs offer a potential solution to this challenge.

In a typical GA, a population of potential solutions is evolved over generations. The solutions, often referred to as chromosomes, are evaluated based on a fitness function that quantifies their quality. The fittest individuals are then selected and modified through crossover and mutation to produce new offspring, and this process continues iteratively until a satisfactory solution is found or a stopping criterion is met.

In the context of trading schedules for share buybacks, the chromosomes could represent different trading strategies or schedules, and the fitness function would be constructed to reflect how well a given strategy performs in replicating observed trading schedules. The use of GAs allows for a flexible, adaptive approach, capable of capturing complex market dynamics and trader behavior.

The selection of GAs for this task is not arbitrary. Their success in various domains, ranging from engineering optimization to machine learning, suggests that they may be well-suited to the financial optimization problems inherent in share buyback trading schedules. Additionally, GAs' ability to handle multimodal and discontinuous search spaces makes them particularly appealing for modeling financial phenomena that may not lend themselves to conventional optimization techniques.

The application of GAs to share buyback trading schedules represents an innovative fusion of computational intelligence and finance. By viewing trading schedules as a search and optimization problem, this hypothesis emphasizes the potential of GAs to provide a deeper understanding of market dynamics and strategic decision-making in share buybacks. It invites further exploration and empirical validation, setting the stage for potentially transformative advancements in the integration of computational methods within financial practice.

**H2: mathematical optimization in share buybacks**

The second hypothesis focuses on the mathematical perspective of share buybacks, considering the buyback process as an optimization problem. Specifically, we hypothesize that the underlying maximization problem in share buyback execution is to maximize the difference between the actual price paid for the shares and the buyback benchmark, represented by the arithmetic average of daily Volume-Weighted Average Prices (VWAPs). This viewpoint aligns the buyback strategy with mathematical optimization principles, offering a rigorous framework for analysis.

Share buybacks are financial transactions where a company purchases its own shares from the open market. This practice can be used for various reasons, such as increasing the value of remaining shares, returning capital to shareholders, or improving financial ratios. The process of determining when and how much to buy is complex and must consider market conditions, regulatory constraints, and the company's financial objectives.

The concept of optimizing share buybacks through mathematical modeling has its roots in classical optimization theory. The mathematical formalization of this hypothesis should take into consideration several factors that contribute to the execution of buyback strategies. These can include, but are not limited to, liquidity constraints, risk management, market impact, and transaction costs. For refer the reader to the seminal paper by Almgren (2003) for some initial considerations.

By representing the share buyback process as a mathematical optimization problem, this hypothesis introduces a structured and quantifiable approach to address these complexities. The objective function to be maximized is the difference between the actual price paid for the shares and the buyback benchmark. Constraints may include regulatory requirements, budget constraints, and market liquidity.

Volume-Weighted Average Prices (VWAPs) play a central role in this hypothesis. VWAP is a trading benchmark used by traders that gives the average price a stock has traded at over a given time frame, weighted by volume. In the context of this hypothesis, the arithmetic average of daily VWAPs serves as a representative measure against which the actual buyback price is compared. Maximizing the difference between the two encapsulates the goal of acquiring shares at favorable prices relative to market trends.

The formulation of this problem necessitates sophisticated mathematical techniques. Various optimization methods could be explored, such as linear programming, nonlinear programming, or even metaheuristic approaches like simulated annealing or genetic algorithms. The selection of the method may depend on the specific characteristics of the problem, such as the nature of the constraints, the presence of multiple objectives, or the existence of uncertainty in market conditions.

This hypothesis not only provides a theoretical foundation for the mathematical modeling of share buybacks but also opens the door to empirical validation and practical implementation. The translation of buyback strategies into a well-defined optimization problem allows for systematic exploration, analysis, and solution, supported by established mathematical and computational tools. It also invites collaboration between financial practitioners and optimization experts, bridging the gap between financial decision-making and mathematical rigor.

The potential implications are significant, impacting both academic research and industry practice. By offering a novel, quantitative approach to share buyback execution, this hypothesis can stimulate further investigation into the underlying dynamics of buybacks, enrich the toolkit available to financial professionals, and contribute to the ongoing evolution of financial optimization and decision-making. It aligns with contemporary trends in finance that emphasize data-driven, analytical methods, reflecting a broader shift toward the integration of mathematical and computational techniques in financial management and strategy.

**H3: optionality in buyback execution**

The third hypothesis explores the concept of optionality in the execution problem of share buybacks. We posit that incorporating optionality, or the ability to dynamically adapt the trading schedule, can substantially increase the outperformance of the buyback benchmark. This hypothesis underscores the importance of flexibility and adaptive strategies in maximizing the effectiveness of buyback operations.

Optionality in financial contexts refers to the ability to make decisions that are contingent on the realization of uncertain future events. In the context of share buybacks, optionality implies the capability of adapting the trading schedule and execution strategy in response to dynamic market conditions, emerging opportunities, and unexpected challenges.

This hypothesis builds on the notion that share buybacks are not merely mechanical processes but involve strategic decision-making, balancing a multitude of factors such as market trends, regulatory constraints, corporate objectives, investor expectations, and operational considerations. The concept of optionality brings a nuanced understanding of how to navigate this intricate landscape, suggesting a more agile and responsive approach to buyback execution.

To elucidate the significance of optionality in buyback execution, we can dissect its multiple dimensions and implications:

- Real-time market adaptation: Optionality enables trading strategies to adapt to real-time market conditions, recognizing short-term trends, liquidity constraints, or sudden market shocks. It facilitates the dynamic adjustment of trading volumes, timing, or pricing to capitalize on transient opportunities or mitigate unforeseen risks.
- Strategic flexibility: By allowing ongoing reassessment and reconfiguration of the trading schedule, optionality supports alignment with broader corporate goals and market strategies. It ensures that buyback execution is not confined to a rigid plan but can evolve in harmony with the shifting corporate landscape.
- Risk management: Incorporating optionality provides a mechanism to actively manage risks associated with share buyback execution, such as market impact, price slippage, regulatory compliance, or adverse market movements. It encourages a proactive stance toward risk, embedding responsiveness and resilience in buyback strategies.
- Enhanced performance metrics: The ability to adapt the trading strategy offers the potential to outperform static benchmarks, driving greater efficiency and effectiveness in buyback execution. Optionality could translate to better price execution, reduced transaction costs, and alignment with best execution practices.
- Technological and analytical integration: Embracing optionality necessitates advanced technology and analytical capabilities to monitor market conditions, evaluate opportunities and risks, and execute real-time adjustments. This alignment with technology under-scores the synergy between optionality and modern trading systems, supporting algorithmic execution and data-driven decision-making.
- Ethical and regulatory considerations: The dynamic adaptation of trading schedules must be executed within the bounds of legal and ethical guidelines. Understanding and managing the interplay between optionality, transparency, and accountability is crucial to maintain integrity and trust in buyback activities.
- Interdisciplinary collaboration: Optionality in buyback execution demands collaboration between various stakeholders, including finance professionals, data scientists, economists, legal experts, and technology developers. It represents a multidisciplinary challenge that invites innovation and integration across these diverse domains.

In conclusion, the hypothesis of optionality in buyback execution extends the traditional paradigm of share buybacks by embedding a dynamic, adaptive dimension. It recognizes the inherent complexity and uncertainty in financial markets, advocating a more flexible, responsive, and holistic approach to buyback execution. This theoretical construct not only contributes to academic discourse but holds tangible relevance for financial practitioners, policy-makers, and technologists. By unearthing the potential of optionality, it opens new horizons for research, development, and implementation in the evolving field of share buybacks, reflecting a contemporary perspective that resonates with the multifaceted nature of modern financial management and trading strategies.

Together, these hypotheses forge a cohesive and innovative theoretical framework, opening up new dimensions in the study of share buybacks. By marrying principles from financial economics, mathematical optimization, and computational intelligence, they invite further exploration and testing, aiming to catalyze transformative advancements in the field of financial innovation.

The synergy of these diverse principles fosters a multifaceted approach to understanding and optimizing share buybacks. Here, we highlight some significant avenues that emerge from this integrated framework:

- Interdisciplinary research opportunities: The convergence of financial economics, mathematical optimization, and computational intelligence encourages interdisciplinary collaboration and research. Scholars, practitioners, and policymakers from various fields can engage in a collective quest to validate, refine, and extend these hypotheses, building a more comprehensive understanding of share buyback strategies.
- Technological innovation: The application of Genetic Algorithms and mathematical optimization to share buybacks presents a fertile ground for technological innovation. These hypotheses provide a blueprint for developing new tools, platforms, and algorithms that can operationalize the theoretical insights, transforming them into actionable strategies for financial managers and traders.
- Strategic impact on corporate finance: By redefining the way share buybacks are conceptualized and executed, these hypotheses hold the potential to influence corporate financial strategies. The insights on trading schedules, mathematical optimization, and optionality can guide corporations in devising more effective, adaptive, and efficient share buyback plans, enhancing shareholder value.
- Regulatory implications: The innovative perspective proposed in these hypotheses may prompt a reevaluation of regulatory frameworks governing share buybacks. Understanding the complexities and potential benefits of these novel approaches could lead to more informed regulatory policies, ensuring that innovation aligns with ethical considerations and market integrity.
- Educational contributions: The theoretical framework provides a rich educational resource, infusing traditional financial curricula with contemporary insights from computational intelligence and mathematical optimization. It can foster a new generation of finance professionals who are equipped with the knowledge and skills to navigate the complex landscape of modern financial markets.
- Global relevance: These hypotheses are not confined to a specific geographic or market context but resonate with the global dynamics of financial markets. They offer a universal perspective that can be adapted and tested across diverse markets and economic systems, contributing to a more coherent and interconnected global financial ecosystem.
- Sustainable financial practices: The integration of advanced algorithms and mathematical techniques may pave the way for more transparent, accountable, and sustainable financial practices. The insights derived from these hypotheses could contribute to the broader agenda of responsible finance, aligning share buybacks with social, ethical, and environmental considerations.

The triad of hypotheses articulated in this article represents more than a mere theoretical exercise; it lays the foundation for a paradigm shift in the field of share buybacks and financial innovation. By intertwining disciplines and connecting theory with practice, these hypotheses illuminate a path forward, igniting curiosity, creativity, and collaboration. They challenge conventional wisdom, provoke critical thinking, and stimulate intellectual exploration, all the while preserving a connection to real-world applications and implications. The blend of rigorous analysis, imaginative hypothesis-building, and strategic foresight contributes to an academic and practical discourse that is poised to shape the future of share buybacks, reflecting a contemporary perspective that resonates with the multifaceted nature of modern financial management and innovation.

## 3. Discussion and implications

The proposed hypotheses collectively signal a groundbreaking shift in the approach to share buybacks, integrating computational methods such as Genetic Algorithms with traditional financial economics. These ideas bear the potential to revolutionize share buyback strategies, offering more dynamic, adaptive, and optimized solutions.

Potential impact: The validation of these hypotheses could lead to more efficient and effective execution of share buybacks, aligning closely with market benchmarks and investor expectations. By employing Genetic Algorithms and mathematical optimization, corporations may find innovative pathways to enhance shareholder value and manipulate capital structure.

Testability: The hypotheses lend themselves to empirical testing through both simulation and real-world application. Computational experiments with Genetic Algorithms can model trading schedules, while mathematical optimization techniques can provide rigorous analysis. In combination with market data (It is one of the research tasks to gather the relevant data from brokers and companies.), these methodologies can offer robust validation or refutation of the hypotheses, fueling further innovation and research.

### 3.1. Extensions to evolutionary algorithms

Evolutionary Algorithms (EAs), embodying the principles of natural evolution, represent a vibrant and diverse family of optimization techniques. While our research primarily employs Genetic Algorithms (GAs), the exploration of alternative EAs warrants attention as a valuable extension.

Particle swarm optimization (PSO): A bio-inspired optimization technique based on the social behavior of bird flocking or fish schooling. PSO could potentially offer advantages in terms of simplicity and speed of convergence. Employing PSO in the context of share buybacks would require extensive analysis to adapt its swarm behavior principles to the dynamics and constraints of financial trading.

Differential evolution (DE): DE, with its mechanism of vector operation, can optimize real-valued multivariate functions and is acknowledged for its simplicity and robustness. The application of DE in our context could navigate through the solution space differently than GAs and PSO, possibly unveiling distinct solution pathways and strategies for share buyback optimization.

Simulated annealing (SA): An optimization method inspired by the annealing process in metallurgy, SA allows for the possibility of accepting less optimal solutions in early iterations to escape local minima. This characteristic could be particularly advantageous in the intricate and potentially rugged solution landscapes encountered in financial optimization problems.

Ant colony optimization (ACO): Inspired by the foraging behavior of ants, ACO leverages a collective behavior to find optimal paths. The strategic moves in share buyback can be viewed as a path in a solution space, where ACO can offer insights into optimized sequential decision-making.

Each of these EAs offers unique characteristics and exploration strategies in the solution space. A comparative study among these algorithms could unearth the respective strengths and weaknesses relative to the complexities of share buyback optimization problems. Thus, future research endeavors could harness these alternative EAs, exploring a richer and more diverse array of solution methodologies to further enhance the efficacy and strategic intelligence of share buyback initiatives.

The hypotheses presented in this article are not merely theoretical musings; they present actionable insights, bridging the gap between academic theory and practical finance. The implications reach beyond share buybacks, suggesting broader applications of computational intelligence in financial decision-making, and opening doors to interdisciplinary research, connecting economics, mathematics, and computer science in pursuit of financial innovation.

This article has introduced novel hypotheses concerning the use of Genetic Algorithms and mathematical optimization in the execution of share buybacks. These hypotheses, grounded in both traditional financial economics and computational intelligence, offer a fresh perspective that may reshape our understanding of share buyback strategies.

The potential impact of these ideas extends well beyond the immediate subject, opening new vistas in the broader field of financial innovation. The testability of the hypotheses invites empirical exploration and may lead to tangible improvements in share buyback execution and other financial operations.

Looking ahead, further research should delve into the detailed mechanisms by which Genetic Algorithms can be tailored to share buyback execution, the mathematical properties governing the optimization problems, and the practical considerations for implementing these theories. Such endeavors promise to refine and expand these initial hypotheses, contributing to a vibrant and evolving field that unites financial theory, computational methods, and practical applications in a synergistic pursuit of innovation.

## Data availability statement

The original contributions presented in the study are included in the article/supplementary material, further inquiries can be directed to the corresponding author.

## Author contributions

JO: Conceptualization, Data curation, Formal analysis, Funding acquisition, Investigation, Methodology, Project administration, Resources, Software, Supervision, Validation, Visualization, Writing—original draft, Writing—review & editing.
